# Defining a Correlative Transcriptional Signature Associated with Bulk Histone H3 Acetylation Levels in Adult Glioblastomas

**DOI:** 10.3390/cells12030374

**Published:** 2023-01-19

**Authors:** Irati Hervás-Corpión, Jorge Navarro-Calvo, Paula Martín-Climent, Marianela Iriarte-Gahete, Noelia Geribaldi-Doldán, Carmen Castro, Luis M. Valor

**Affiliations:** 1 Unidad de Investigación, Hospital Universitario Puerta del Mar, INiBICA, 11009 Cádiz, Spain; 2 Programa de Tumores Sólidos, Centro de Investigación Médica Aplicada (CIMA), Clínica Universidad de Navarra, 31008 Pamplona, Spain; 3 Unidad de Investigación, Hospital General Universitario Dr. Balmis, ISABIAL, 03010 Alicante, Spain; 4 Servicio de Inmunología, Hospital Universitario Puerta del Mar, 11009 Cádiz, Spain; 5 Facultad de Medicina, Universidad de Cádiz, INiBICA, 11003 Cádiz, Spain

**Keywords:** glioma, epigenetics, histone, acetylation, RNA-seq, HDACi, TSA

## Abstract

Glioblastoma (GB) is the most prevalent primary brain cancer and the most aggressive form of glioma because of its poor prognosis and high recurrence. To confirm the importance of epigenetics in glioma, we explored The Cancer Gene Atlas (TCGA) database and we found that several histone/DNA modifications and chromatin remodeling factors were affected at transcriptional and genetic levels in GB compared to lower-grade gliomas. We associated these alterations in our own cohort of study with a significant reduction in the bulk levels of acetylated lysines 9 and 14 of histone H3 in high-grade compared to low-grade tumors. Within GB, we performed an RNA-seq analysis between samples exhibiting the lowest and highest levels of acetylated H3 in the cohort; these results are in general concordance with the transcriptional changes obtained after histone deacetylase (HDAC) inhibition of GB-derived cultures that affected relevant genes in glioma biology and treatment (e.g., *A2ML1, CD83*, *SLC17A7, TNFSF18*). Overall, we identified a transcriptional signature linked to histone acetylation that was potentially associated with good prognosis, i.e., high overall survival and low rate of somatic mutations in epigenetically related genes in GB. Our study identifies lysine acetylation as a key defective histone modification in adult high-grade glioma, and offers novel insights regarding the use of HDAC inhibitors in therapy.

## 1. Introduction

Gliomas constitute a heterogenous group of primary brain tumors that include the especially aggressive glioblastomas (GB) [1]. Current treatments are primarily based on the application of the Stupp protocol, which consists of surgical resection of the tumor mass, radiotherapy, and chemotherapy with temozolomide (TMZ) [2]; however, they are still insufficient to avoid recurrence in nearly all cases. Epigenetics dysregulation can be relevant in the formation and maintenance of GB because glioma stem cells, which are thought to be responsible of GB recurrence, are maintained in an undifferentiated and self-renewal state due to a permanent epigenetic block [3]. Thus, the epigenetic landscape of the tumoral mass can have a significant impact on the patient outcome. For example, the silencing of the O-6-methylguanine-DNA methyltransferase (*MGMT*) gene by hypermethylation of its promoter improves the efficacy of alkylant agents such as TMZ [4]. Moreover, the most important molecular alteration with diagnosis utility to date, the mutation of arginine 132 in the isocitrate dehydrogenase (*IDH1*) gene, is sufficient to remodel the DNA methylome [5], a phenomenon that may explain the better outcomes in mutated gliomas compared to wild type tumors.

Among the covalent modifications involved in glioma epigenetics, special attention has been paid for lysine acetylation of histone tails because it can be modulated by several compounds belonging to different chemical families. Histone acetylation is regulated by the opposing activities of lysine acetyltransferases (KAT) and histone deacetylases (HDAC); histone deacetylation is associated with more compact chromatin and silent/repressed genes, and increased levels of histone acetylation are associated with a more relaxed chromatin and active genes. Disruption of HDAC activity has been widely documented in cancer, in which several HDAC inhibitors (HDACis) have been tested alone or as part of combined therapies [6]. HDACis presumably can derepress the expression of tumor suppressor genes but can also facilitate the chromatin accessibility for DNA damaging agents [7], acting as sensitizers of the conventional treatments [8,9]. In glioma cell lines, HDACis perform antiproliferative and apoptotic actions [10,11] by regulating genes that control cell death and cell cycle [6]. As an example, suberoylanilide hydroxamic acid (SAHA, also known as vorinostat) induces the histone H3 acetylation of the p21/*CDKNA1* gene promoter, concomitant with the upregulation of the gene [12], plausibly by providing relief to the deacetylation action of HDAC4 at this promoter region [13]. Although several HDACis have been tested, their high toxicity and low specificity have prevented their applicability into the clinical management of patients [14,15,16]. For example, SAHA is also able to upregulate the expression of *MGMT* to potentiate the chemoresistance for TMZ treatment [17]; trichostatin A (TSA) can induce the hyperacetylation of H3K9 at the promoter II of the *glial cell line-derived neurotrophic factor* (*GDNF*) gene [18], a factor that is tightly related to gliomagenesis [19]; in epidermal growth factor receptor (EGFR)-driven gliomas, the dissociation of HDAC3 from the *cyclin D1* (*CCDN1*) and *MYC* promoters increases H3K9 acetylation at these regions and leads to the transcriptional activation of these proto-oncogenes to promote cell proliferation and tumorigenesis [20,21], a phenomenon that can accelerated by HDACi treatment. In the absence of pharmacological manipulation, basal histone acetylation may also influence the clinical outcome of gliomas, as suggested in reported immunohistochemistry assays: low-grade tumors showing < 88% of H3K9ac^+^ cells were more likely to be associated with a reduced survival, whereas GB showing < 74% of H3K18ac^+^ cells were significantly associated with longer overall and progression-free survival [22]. Overall, we need to understand the role of histone acetylation in GB to refine epigenetic-based prognosis biomarkers and therapeutic strategies.

## 2. Materials and Methods

### 2.1. Processing of Human Samples for Western Blotting, RT-qPCR, and RNA-seq Assays

Samples were obtained from glioma surgical resections according to the standard procedures proposed by the Sistema Sanitario Público de Andalucía (SSPA) Biobank of the Hospital Universitario Puerta del Mar (HUPM, Cádiz, Spain). Table 1 summarizes the main characteristics of the cohort used in the present study.

Samples were processed following the procedures described in [23] for Western blotting and retrotranscription-quantitative PCR (RT-qPCR) assays and RNA-seq analysis. For Western blotting normalization, we first normalized the signal intensities of the band with the signal intensity of the same external protein extract loaded in all gels; we next normalized the values of each histone modification with the corresponding value of total histone H3 per sample, as we were interested on the fraction of the total protein with such covalent modification independently on potential variations of total histone H3 across gliomas. Samples with low signal intensity for total histone H3 were discarded. Western blotting assays were performed using the same cohort previously reported in [23], adding 7 GBs not included at the time in these assays. For RT-qPCR, we used TBP as the housekeeping gene. For Western blotting, the following antibodies were used: H3K9,14ac (06-599), H3K4me3 (07-473), H3K27me3 (07-449) (1:1000, EMD Millipore, Darmstadt, Germany), total H3 (1:8000, ab1791, Abcam, Cambridge, UK), and HRP-conjugated secondary antibodies (1:7500, A0545 and A4416, Sigma-Aldrich, Darmstadt, Germany). The sequences of primer pairs used in the RT-qPCR assays are listed as follows: A2ML1, 5′-CATTGTTGGCCCAGCTTACC-3′ and 5′-ATGTGCGCTGGAAATTCTCAG-3′, CD83, 5′-GAGGGTGGTGAAGAGAGGATG-3′ and 5′-CTCTTCTTTACGCTGTGCAGG-3′, CDYL2, 5′-CTCAGATACA- GTGTCCGCCAG-3′ and 5′-TCGCCGGACTTCTTTCATGAT-3′, GABRA4, 5′-GATGGTCATGCATGCCCTTTG-3′ and 5′-ACTTGATACGGTTTGCCCAATC-3′, GFAP, 5′-AGAGGAAGATTGAGTCGCTGG-3′ and 5′-TGTCAGGTCTGC- AAACTTGGA-3′, HPCAL4, 5′-GAAGCTCAACTGGGCCTTTG-3′ and 5′-GGTCGTCCTTATCCTGGTCC-3′, PROM1 (CD133) 5′-ACTCAGCGTCTTCCTATTCAG-3′ and 5′-AAAATCACGATGAGGGTCAGC-3′, SLC17A7, 5′-GCTTCGGGATCTTCTGGTACC-3′ and 5′-CAGAAGTTGG- CCACGATGATG-3′, SLITRK4, 5′-AATCCTGACTGTGGCTCCAT-3′ and 5′-ATCTTTCTCCTTGTCGGCCA-3′, TNFSF18, 5′-TCTTTGCTCCTTCAGTTGGC-3′ and 5′-ATACAGCCGCACCTCAAAAG-3′. The sequences of TBP primers are described in [23].

### 2.2. DNA Methylome Analysis

In total, 8 GBs were randomly selected for this analysis: 6 females and 2 males with a median of age of 62.5 years (IQR = 11.25). Bisulfite-treated DNA from these samples was labelled and hybridized into Infinium MethylationEPIC beadchips (Illumina, San Diego, CA, USA) according to the manufacturer’s recommendations at the Unidad de Genómica of Genyo (Granada, Spain).

The “minfi” software was used for data extraction, quality assessment, background correction with dye bias normalization (using the method of normal–exponential out-of-band or Noob, implemented in the package), and the filtering of probe sets located in SNPs and sexual chromosomes [24]; β-values (methylated Cy5/unmethylated Cy3 signals) were normalized using the BMIQ method implemented in the “RnBeads” R-based package [25], and the CpG sites were mapped into the GRCh38/hg38 genome assembly at the Unidad de Bioinformática of Genyo. Differential methylation was determined using the “limma” package [26]. We only considered the CpG sites that were mapped into the genes interrogated in the RNA-seq analysis, resulting in 13,354 genes containing both transcriptomics and epigenomics data in our cohort of study.

### 2.3. Primary Glioblastoma Cultures

For culturing, a glioblastoma sample from a surgical resection was dissociated enzymatically and the cells were maintained as neurospheres in DMEM/F-12 medium supplemented with B27 and N2 (Gibco, Thermo Fisher, Madrid, Spain) plus epidermal growth factor (EGF) and basic fibroblast growth factor (bFGF) (10 ng/mL, PeproTech, London, UK) [27]. Seven days later, neurospheres were attached to a laminin-coated flask using serum-containing medium [28]. These cultures were established in conditions that favored the proliferation and maintenance of glioma stem cells [28], while minimizing the heterogeneity of resected tissues and enabling their pharmacological manipulation. Subsequently, cells were seeded in 12-well plates (5 × 10^4^ cells/well) and treated with 2 µM TSA (Sigma-Aldrich, Darmstadt, Germany) for 24 h. After treatment, cell viability was assessed using the XTT cell proliferation assay according to manufacturer’s instructions (Canvax Biotex, Valladolid, Spain) and cells were processed for downstream experiments using the same procedures as tissue resections [23]. The only modification concerned RNA-seq analysis in which DNA libraries were obtained using the TruSeq Stranded mRNA kit (Illumina, San Diego, CA, USA) and sequenced in a 75 bp paired-end configuration (Unidad de Genómica, Cabimer, Sevilla, Spain).

### 2.4. Additional Statistical Analysis and Bioinformatics

Mann-Whitney U tests, Student’s *t*-tests, Pearson’s product moment correlations, χ^2^ test, and heatmap plotting were performed using the native R environment. Principal component analysis (PCA) was conducted with the “rgl” package (http://cran.r-project.org/package=rgl) (accessed on 6 June 2022), and survival analysis using the “survminer” and “survival” packages to calculate both the Kaplan–Meier curves and log-rank *p*-values (accessed on 20 October 2022) in the R environment (R version 4.0.5). In this analysis, we divided the samples into three parts after trial and error, and compared the days to death associated with the third of data containing the highest expression values and the third of data containing the lowest expression values for each dataset.

We used the genetics and transcriptomics information from The Cancer Gene Atlas (TCGA) consortium as deposited in the Genomic Data Commons (GDC) website (https://portalgdc.cancer.gov, accessed on 6 June 2022), and from the REMBRANDT cohort as deposited in GeneBank with the accession number GSE108474. Differential expression between GB and lower-grade glioma (LGG) datasets was previously calculated [23,29].

Epigenetic genes were obtained from the EpiFactors database [30], which included enzymes and associated cofactors related to epigenetic regulation. Of the 815 proteins contained in the database, we only considered 590 related to histone modification (acetylation, methylation, phosphorylation, and ubiquitylation), DNA modification, chromatin remodeling, and readers, either containing enzymatic activity or acting as cofactors, after excluding histones, protamines, chaperones, epitranscriptomics and unclassified factors.

## 3. Results

### 3.1. Histone Acetylation Levels Is Defective in Glioblastoma Compared to Lower-Grade Gliomas

**Figure 1:**
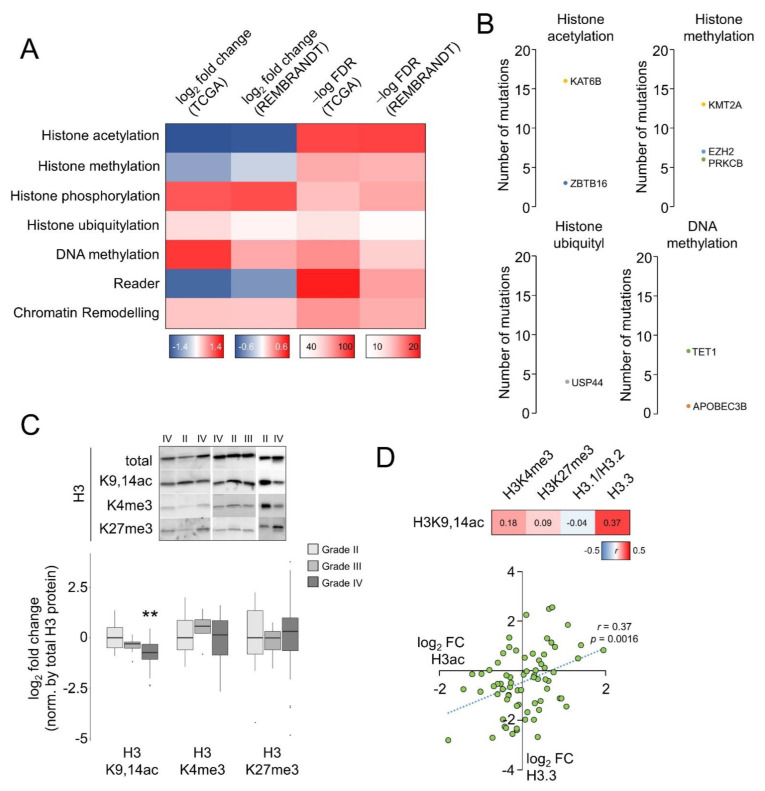
Histone H3 deacetylation in glioblastomas: (**A**) Genes with differential expression between the datasets of “GBM” and “LGG” projects of TCGA that were also confirmed in the REMBRANDT cohort. Individual values of fold change and adjusted *p*-values are shown in Figure S1. FDR, false discovery rate. (**B**) For the number of somatic mutations, we only consider nonsynonymous substitutions in protein coding regions in the differentially expressed genes of (**A**). (**C**) Upper panel, representative Western blots for total and covalently modified histones in tumor resections where each lane represents a patient’s sample; the number denotes the histological grade. Lower panel, quantification of the blots after normalization by the total H3 signal. *n* = 13 for grade II, *n* = 7 for grade III, *n* = 57 for grade IV (GB). **, *p*-value < 0.005, Mann–Whitney U test related to grade II. Raw blots are shown in Figure S2 with the location of the image crops. (**D**) Pearson correlation coefficients (*r*) for H3K9,14ac levels and other histone modifications (this study) and histone H3 variants [23]. The plot shows the correlation between H3K9,14ac and H3.3, the only one to be significant.

In an attempt to explain glioma malignancy, we compared the gene expression profiles between GB and LGG using the transcriptomics datasets contained in the GDC portal (hereafter referred to as TCGA datasets), under the assumption that the differential expression between these two major types of glioma might identify the key factors that enhance the characteristic aggressivity of GB. This analysis revealed an extensive differential expression: ~23% of the whole transcriptome (fold change > 2, adjusted *p*-value < 0.05), which affected ~11% of genes related to DNA and histone modifiers and readers, chromatin-remodeling genes, and cofactors according to the curation of EpiFactors [30] (hereafter referred to as epigenetic genes). Of these, genes modulating histone acetylation were more consistently affected because they exhibited the largest and significant differential expression, according to the absolute averages of fold change and adjusted *p*-values for the common genes also differentially expressed in the REMBRANDT cohort (adjusted *p*-value < 0.05) (Figure 1A and Figure S1). In addition to the gene expression variations, we also observed a slightly higher rate of somatic mutations in these histone acetylation regulators in the TCGA gliomas compared to other epigenetic categories; *KAT6B* was the only gene containing the same mutation (T1203Rfs*21) at a single position in more than one case (four cases), but no mutated genes were found in “histone phosphorylation”, “reader”, or “chromatin remodeling” categories (Figure 1B). Overall, this analysis indicates that enzymes and cofactors regulating histone modifications, especially histone acetylation, were affected in gliomas and might be linked to relevant clinical outcomes.

To determine whether these alterations correlated with effective changes in histone modifications, we performed Western blotting assays in two collections of glioma surgical resections that are described in our previous work [23]: after scanning the bulk levels of well-known covalent modifications of histone H3 in tumor resections, only the acetylated lysines 9 and 14 (H3K9,14ac or H3ac) were significantly different between GB and grade II gliomas after normalizing by total histone H3 levels; grade III represented an intermediate state, although caution should be taken in this interpretation because of the low number of samples and the sex bias. For simplicity, Figure 1C shows the combined results for both cohorts. However, the bulk levels for trimethylation of either H3K4 or H3K27 were not significantly different across grades (Figure 1C) and did not correlate with the levels of acetylated histone H3 (Figure 1D). In contrast, the bulk levels of the replication-independent variant H3.3, also reduced in adult high-grade gliomas [23], were significantly correlated with H3K9,14ac signal intensities (Figure 1D), suggesting that H3.3 was acetylated at these lysines.

### 3.2. A Transcriptional Signature Can Be Correlated with Differential Bulk Levels of Histone Acetylation in Glioblastoma

**Figure 2:**
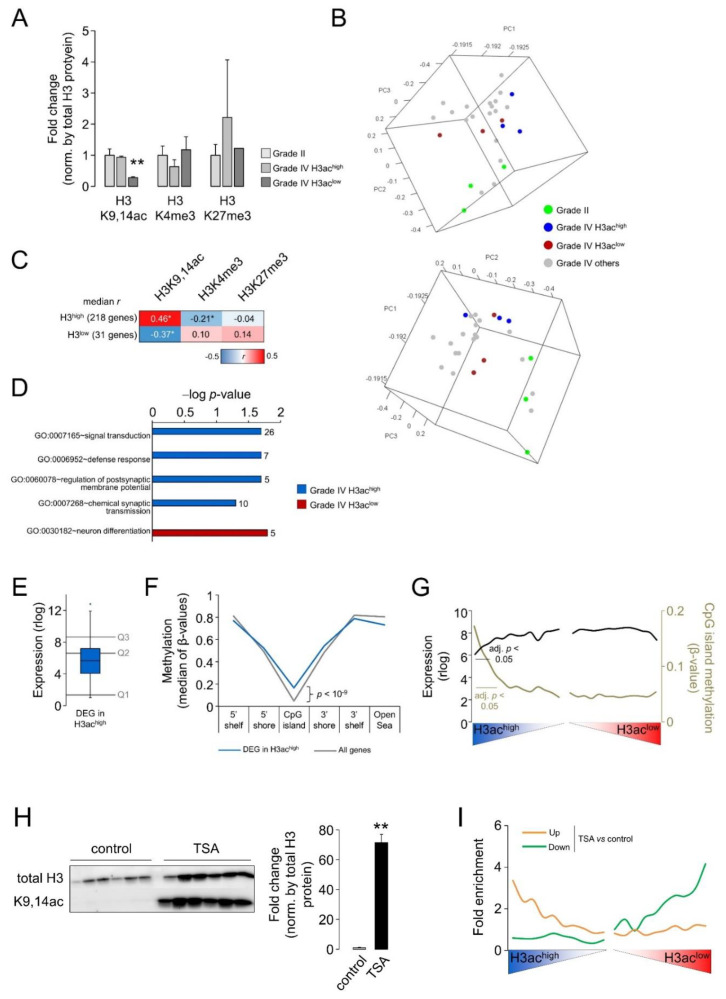
Transcriptional signatures associated with differential levels of histone H3 acetylation in glioblastomas: (**A**) H3K9,14ac levels in the selected samples for the differential expression analysis; GBs expressing lower and similar levels of acetylated protein compared to grade II gliomas (H3ac^low^ and H3ac^high^, respectively). The data are expressed as the mean ± SEM. **, *p*-value < 0.005; Mann–Whitney U test between both types of GB. (**B**) Tridimensional PCA of the whole transcriptomes for selected gliomas of our cohort, in which RNA was optimal for deep sequencing. (**C**) Median of the Pearson correlation coefficients (*r*) between the expression of each gene in the 249-gene signature (divided upon direction of change) and the bulk level of each histone modification. *, *p*-value < 0.05. (**D**) Significantly enriched GO terms (FDR < 0.05, DAVID) in the DEG between H3ac^low^ and H3ac^high^; the plot shows −log-adjusted *p*-value (bars) and number of genes (next to each bar) for each GO term. (**E**) Box-and-whisker plot of the average expression across GB of the H3ac^high^-signature. Quartiles of expression of the whole GB transcriptome are shown. (**F**) Average CpG methylation expressed as β-values (0, fully unmethylated; 1, fully methylated) of the H3ac^high^-signature compared to the whole GB transcriptome, and divided in CGI, shores (<2 kb from CGIs), shelves (>2 kb and <4 kb from CGIs), and open seas (>4 kb from CGIs). The *p*-value < 0.05 was calculated using Student’s *t*-test between the signature and the rest of genes. (**G**) The GB transcriptome was ranked according to the significance and direction of the gene expression change in the pairwise comparison between H3ac^high^ and H3ac^low^ and divided into bins of 500 genes; median values were calculated for each bin. Adjusted *p*-values were calculated using Student’s *t*-test with Bonferroni correction between each bin of genes and whole population of genes; the plot indicates all significant *p*-values grouped in the genes showing relative higher expression in H3ac^high^ compared to H3ac^low^ GBs. (**H**) Left panel, Western blots for total H3 and H3K9,14ac from untreated and treated GB-derived cultures with TSA. The raw blot is shown in Figure S3. Right panel, quantification of the blots after normalization by total H3 signal. The data are expressed as the mean ± SEM. *n* = 6 for each condition. **, *p*-value < 0.005, Mann–Whitney U test related to the control (vehicle) condition. (**I**) Distribution of DEGs obtained in the pairwise comparisons between TSA-treated and untreated cultures across the whole GB transcriptomes, ordered as in (**G**) and divided into bins of 1000 genes. The number of down- and upregulated genes from our lists was counted in each bin and fold enrichment was calculated (number of observed genes/number of expected genes if equally distributed).

Our Western blotting results reveal general histone hypoacetylation in high-grade tumors. To gain further insights regarding the biological consequences of such alteration in GB, we selected the three GBs showing the lowest and highest levels of H3K9,14ac for which RNA was available in our cohorts, namely, H3ac^low^ and H3ac^high^ (Figure 2A). No differences were observed for bulk levels of trimethylation of K4 or K27 between the selected samples (Figure 2A). In the PCA analysis, these two types of GB were similarly separated from low-grade gliomas despite the comparable bulk histone acetylation levels between H3^high^ GB and low-grade gliomas. This result suggests that different levels of bulk histone H3 acetylation did not have a profound impact on the GB transcriptomes (Figure 2B). After performing differential expression analysis (adjusted *p*-value < 0.05), we identified 249 differentially expressed genes (DEGs) between H3ac^high^ and H3ac^low^ resections, in which 218 genes (~88%) and 31 genes were enriched in H3ac^high^ and H3ac^low^ tumors, respectively (Table S1). This 249-gene signature was specifically associated with H3K9,14ac levels, as this mark showed better correlation coefficients with the median of gene expression than the other histone modifications analyzed in this work (Figure 2C). In the genes enriched in H3ac^low^ GB, we found significant terms for regulation of neurogenesis and neuronal differentiation (e.g., *LHX2*, *FZD8*, *GPC2*, *INSM1*), whereas mature neuronal markers were observed in H3ac^high^ GB that encoded for synaptic proteins (e.g., *SYT1*, *SYN2*) and neurotransmitter receptors (e.g., GABA receptor subunits, *GRM1*; Figure 2D), suggesting that bulk levels of H3K9,14ac might indicate divergent stages of neuronal differentiation and different abundance of healthy neurons within resections.

To understand why some particular genes showed an expression correlated with bulk tumoral levels of H3K9,14ac, we examined whether their gene expression differed from the general GB transcriptome. In addition, we also used information regarding DNA methylation to infer the general epigenetic status of these genes: we randomly selected some samples from our cohort to hybridize bisulfate-treated DNA into Infinium MethylationEPIC beadchips. We did not explore the binding of acetylated histone to DNA, as previous work reported that differential gene expression and differential histone acetylation occupancy across gliomas converged in a low number of common genes [31]. Furthermore, indirect effects on gene expression cannot be disregarded in our histone acetylation-dependent transcriptional signature; therefore, we reason that DNA methylation might be suitable to recapitulate the epigenetic status of the loci of interest. To this aim, we averaged the expression/methylation values of each gene that were common to the RNA-seq and DNA methylation arrays, and we compared the median values of the enriched genes in H3ac^high^ GB with those of the whole population; the number of enriched genes in H3ac^low^ GB were too low to extract meaningful conclusions. The genes associated with high levels of histone acetylation were mostly expressed below the median of gene expression in GB (Figure 2E), and, according to this low expression, were more methylated at their CpG islands (CGIs) (Figure 2F); this is not unsurprising because CGIs usually colocalize within promoters, and have been more tightly linked with the regulation of transcription [32]. Despite their increased methylation, these CGIs still exhibited the expected CpG hypomethylation compared to other genomic features such as shores, shelves, and open seas (Figure 2F). However, this analysis was restricted to the significance cutoff used to define DEGs, probably missing general patterns that might comprise more genes, i.e., although not individually significant, they might be part of changes affecting gene subpopulations. Therefore, we investigated the median values of both gene expression and CGI methylation in windows of 500 genes across the whole GB transcriptomes once arranged according to significance and direction of change, as retrieved in the pairwise comparison between H3ac^high^ and H3ac^low^ GBs. In this manner, we revealed a general extended association between gene responsiveness to elevated histone acetylation at lysines 9 and 14 and low basal gene expression and CGI hypermethylation (Figure 2G), suggesting that well-expressed genes did not need to increase their histone acetylation state to increase their rate of expression [33].

To confirm that the differential levels of H3ac between GBs were linked to the reported transcriptional variations in the RNA-seq analysis, we manipulated H3ac levels by pharmacological means. To this end, we inhibited HDAC activity in GB-derived cultures. Treatment with 2 µM of the HDACi TSA for 24 h resulted in a striking induction of histone hyperacetylation (Figure 2H and Figure S3A) without significant affectation in the viability of cells: untreated, *n* = 6, fold change = 1.00 ± 0.28; TSA-treated, *n* = 6, fold change = 1.23 ± 0.21; SDS-treated, *n* = 2, fold change = 0.00 ± 0.24 in XTT assays. This timing for RNA sampling enabled the identification of the transcriptional changes associated with the TSA treatment prior to any potential cell loss that might introduce bias in the results. In these conditions, the RNA-seq between treated and control cells revealed an extensive transcriptional rearrangement consisting of 1995 and 1261 upregulated and downregulated genes, respectively (Table S2); we validated the upregulation of selected genes in independent treated cultures (Figure S3B). Among the DEGs, we noticed the enrichment of downregulated genes related to cell division and mitosis, and the enrichment of upregulated genes related to synaptic transmission (Figure S3C); the latter result is reminiscent of the GO enrichment documented in the genes that were highly expressed in H3ac^high^ tumors. We also observed an important fraction of epigenetically related genes among DEGs. In contrast, TSA upregulation only affected the expression of ~2.9% epigenetic genes; this percentage was increased to ~11.0% within downregulated genes with TSA, probably as part of a homeostatic response against the pharmacological treatment. These results suggest that HDAC inhibition might have relevant consequences in the reconfiguration of the GB epigenetic landscape. Importantly, these DEGs behaved similar to genes in H3ac^high^ and H3ac^low^ tumors: we plotted this subset of genes across the whole GB transcriptome, once ordered according to the significance and direction of change as a result of the pairwise comparison between H3ac^high^ and H3ac^low^, and we observed that the genes upregulated with TSA tended to be predominantly enriched in the H3ac^high^ GB, whereas the genes downregulated after the treatment tended to be enriched in H3ac^low^ GB (Figure 2I) (χ^2^ = 754.94, d.f. = 19, *p*-value < 0.00001 for downregulation; χ^2^ = 533.38, d.f. = 19, *p*-value < 0.00001 for upregulation).

### 3.3. The Transcriptional Signatures Associated with Histone Acetylation Can Be Linked to Differential Overall Survival Only in the Samples Showing the Most Extreme Expression Levels

When considering adjusted *p*-value cutoffs (<0.05), we found a significant overlap of 33 genes enriched in H3ac^high^ that were also upregulated with TSA treatment (χ^2^ = 29.01, d.f. = 1, *p*-value < 0.00001, Tables S1 and S2). Among them, we found the following genes that have been reported in previous studies: *alpha-2-macroglobulin-like 1* (*A2ML1*), in which protein levels are decreased after in vitro treatments of glioma cells lines [34,35,36]; *CD83*, a dendritic cell maturation marker in which expression is reduced within glioblastoma microenvironment [37,38]; *dedicator of cytokinesis 8* (*DOCK8*), which is associated with progression from low-grade to higher-grade gliomas, and can be lost in chromosome imbalances [39]; *gamma-aminobutyric acid type A receptor subunit alpha 4* (*GABRA4*), a potential inhibitor of cancer progression that is downregulated across glioma grades and negatively correlated with inflammation [40]; *hippocalcin-like 4* (*HPCAL4*), an unfavorable risk factor defined as a core gene in gliomagenesis that becomes upregulated in GB compared to LGG [41,42]; *myocyte enhancer factor 2C* (*MEF2C*), which acts as an oncogene in glioma cells by activating the JAGGED-NOTCH pathway [43]; *solute carrier family 17 member 7* (*SLC17A7*), a tumor suppressor gene with bivalent properties (with co-occupancy of H3K4me3 and H3K27me3 marks) that is overexpressed in patients with glioma-related seizures and downregulated in oligodendrogliomas [44,45,46]; *SLIT and NTRK-like family member 4* (*SLITRK4*), which exhibits a complex expression patterns across brain tumors [47]; *synaptotagmin 1* (*SYT1*), which is defined as a hub gene in GB, associated with poor prognosis and copy number variation [41,47,48]; and *TNF superfamily member 18* (*TNFSF18*), which is moderately beneficial as part of immunotherapy approaches in murine gliomas [49]. In conclusion, high levels of H3K9,14ac might be simultaneously associated with genes with poor and good outcomes.

**Figure 3:**
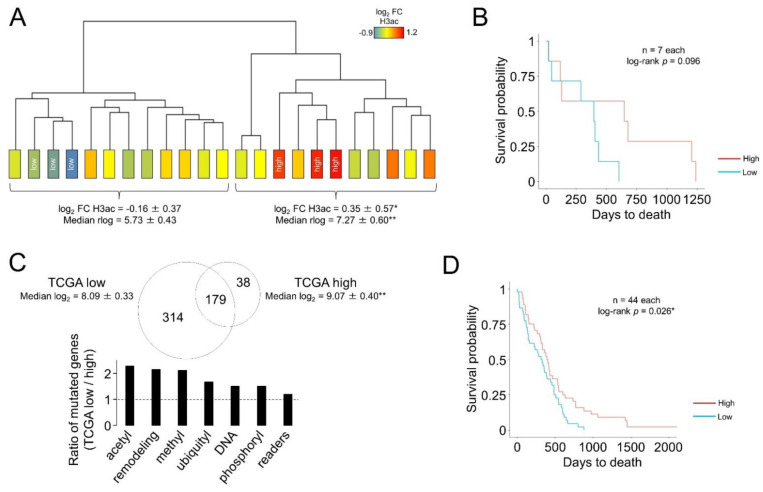
The transcriptional signature linked to bulk levels of histone H3 acetylation as a glioblastoma classifier: (**A**) Hierarchical clustering of our GB cohort using the 33-gene signature (common genes between H3ac^high^ and TSA induction, adjusted *p*-value < 0.05). Log_2_ FC H3ac, fold change in bulk H3ac from the Western blotting analysis of Figure 1. Samples used in the RNA-seq analysis are denoted as “high” (H3ac^high^) and “low” (H3ac^low^). Below, means ± SD of H3ac levels and expression levels of the 33-gene signature of the two segregated groups. *, *p*-value < 0.05, **, *p*-value < 0.005, Mann–Whitney U test between both main clusters. (**B**) Kaplan–Meier curve with associated log-rank *p*-value comparing the probability of survival for the patients of our cohort showing the most extreme values for the median of expression for the 33-gene signature. (**C**) Upper panel, Venn diagram of the genes containing somatic mutations in the two groups of GB from the TCGA database divided according to the overall expression of the 33-gene signature (expressed as mean ± SD). **, *p*-value < 0.005, Mann–Whitney U test between both groups. Lower panel, ratio of the somatic mutations found in each TCGA group for epigenetically related genes. (**D**) The same as in (**B**), using the transcriptional information of the TCGA cohort. *, log-rank *p*-value < 0.05.

However, this subset of 33 might have a net prognostic value as a whole. First, the median expression of this subset enabled the classification of our cohort into two main branches as highly and weakly acetylated tumors (Figure 3A), confirming that this signature was tightly dependent on histone H3 acetylation. However, both groups were not different in terms of overall survival (log-rank *p*-value = 0.5); only when comparing those samples exhibiting the most differential expression values, the median expression of the 33-gene signature had a trend towards a good outcome (Figure 3B). The same approach was unsuccessful using the genes enriched in H3ac^high^ (218-gene signature) and H3ac^low^ (31-gene signature), as we obtained log-rank *p*-values of 0.22 and 0.35, respectively. Taking the TCGA samples for which the “days to death” parameter was available, we used the 33-gene signature to divide the GB into two groups: TCGA-subset^low^ and TCGA-subset^high^ (Figure 3C). The former subset (TCGA-subset^low^) accumulated more somatic mutations than the TCGA-subset^high^ GBs, in which genes involved in the regulation of histone acetylation, followed by histone methylation and chromatin remodeling, were more frequently mutated (Figure 3C), suggesting a relationship between somatic mutation of the epigenetic enzymatic machinery and the altered gene expression patterns of the H3ac-linked signature. Independent of this classification, the median expression of the 33-gene signature in the TCGA database was significantly associated with a good prognosis after comparing samples containing the most extreme values (Figure 3D).

## 4. Discussion

In this work, we describe the transcriptional and genetic alterations that affect epigenetic regulators in GB. Such alterations may influence the tumoral epigenetic landscape with potential consequences for survival, quality of life, and response to treatment. By comparing overall modifiers according to their type of epigenetic activity (DNA and histone modifications, readers of these modifications, and chromatin remodeling factors), we observed that the most consistent alterations occurred in genes controlling histone acetylation, independent of whether they participate in writing or erasing this post-translational modification. More attention has been paid for HDAC, reporting either decreased or increased gene expression levels across histological grades and normal tissue [50,51,52,53]. In agreement with references [50] and [53], we found the downregulation of *HDAC4* and *HDAC11* in GB to be related to LGG. The class II HDAC4 was found to inhibit the expression of the proapoptotic p21/*CDKN1A* gene [13], the high expression levels of which may have a poor prognostic value in the GB mesenchymal subtype [54], and may promote radioresistance [55]; therefore, the general downregulation in GB of TCGA and REMBRANDT cohorts compared to LGG may probably be part of a homeostatic response to refrain tumor malignancy. Apart from a recent study suggesting a role in glioma progression and therapeutical resistance [56], little is known about the role of *KAT6B* in glioma, a lysine acetyltransferase of the MYST family with oncogenic or tumor suppressor properties depending on the cancer [57]. However, the KAT6B activity seems to be more critical in regulating the acetylation of lysine 23 [58], and cannot explain, in principle, the decreased levels of bulk H3K9,14ac in our samples. Overall, complex interactions between the altered activities of KAT, HDAC, and cofactors may result in a net balance of H3K9,14ac reduction in GB.

Although gliomas showed increased histone H3 acetylation levels compared to normal brain tissue in a Western blotting analysis, no conclusive result was obtained when comparing gliomas of different grades in a previous report [50]. In our work, we were able to observe decreased levels of bulk histone H3 acetylation in GB resections compared to low-grade gliomas. Of note, such reduction paralleled the levels of H3.3 in our previous work using the same extract proteins [23]. Interestingly, the mutation of *H3-3A* (also known as *H3F3A*) in pediatric high-grade gliomas interferes with the activity of EZH2 methyltransferase/PRC2 complex, leading to an overall decrease in the levels of H3K27me3 concomitant to an increase in H3K27ac [59]; the observation for such hyperacetylation was extended to other histone lysines [60]. According to these results, it seems that the depletion of wild type H3.3 in adult gliomas (causing a loss of function [61]) associates with histone deacetylation, whereas the H3.3 mutation in pediatric gliomas (causing a gain-of-function) is linked to histone hyperacetylation.

However, differential bulk levels in H3K9,14ac were not sufficient for a profound differential gene expression among GB, suggesting that additional histone covalent modifications (or alternative histone acetylation marks, such as H3K27ac, a mark that is part of transcriptional activation domains in the glioma chromatin [31,33]) are required for understanding the GB transcriptome arrangement. However, we observed that genes with low (but not absent) expression were more likely to exhibit variation in the levels of H3K9,14ac, suggesting that an increase in H3ac does not necessarily influence the transcription of highly expressed genes, as they might already contain sufficient acetylated histones at their regulatory regions. This fact should be considered regarding the use of HDACis (or alternatively, KAT enhancers) to manipulate the expression of tumor suppressor genes. Nonetheless, most well-known HDACis (such as TSA) are relatively unspecific and inhibit entire classes of HDAC that not only regulate histones, but also transcription factors that may also affect gene expression; additionally, histone-independent actions should not be disregarded [3]. Such wide actions may explain contradictory outputs regarding the induction of genes of good and poor prognosis simultaneously (see examples in the Results section), despite the downregulation of mitosis- and proliferation-related genes. This phenomenon may result in ineffective actions over the tumoral mass in failed clinical trials, in addition to the poor pharmacokinetics of most HDACis [15,16]. Moreover, HDACi treatment evokes a complex homeostatic response (e.g., resulting in the downregulation of regulatory genes with opposite actions such as *KAT6A* and *HDAC10* in our cellular preparations; Table S2) that may be key to understanding the induced chemoresistance against these epigenetic drugs after long-term regimes [17,62]. Therefore, functional assays are required to assess the tumorigenic effects of candidates. Furthermore, chromatin immunoprecipitation will determine the direct targets that will be helpful for the design of more selective compounds for pharmacological modulation of histone acetylation within the signature. Overall, knowing the precise targets of these epigenetic inhibitors is crucial in predicting the clinical outcome after treatment and in improving therapeutics in patients.

## Figures and Tables

**Table 1:** Clinical characteristics of the samples used in the present study. Refer to the Supplementary Table S1 of ref. [23] for a description of the samples (age, sex, diagnosis, histological grade, location of each tumor). IQR, interquartile range.

Grade	Sex	Number of Samples	Median (Age)	IQR (Age)
II	Female	5	44	9
	Male	8		
III	Male	7	47	11.5
IV	Female	29	62	18
	Male	28		

## Data Availability

In-house datasets generated and analyzed during the current study are available in the GEO repository under accession numbers GSE147391 and GSE185861.

## Supplementary Materials

The following supporting information can be downloaded at https://www.mdpi.com/article/10.3390/cells12030374/s1. Figure S1: Differential expression changes in epigenetically related genes in TCGA and REMBRANDT gliomas. Figure S2: Raw Western blots of histone H3 modifications in glioma resections. Figure S3: Histone H3 acetylation in TSA-treated glioma culture. Table S1: Complete RNA-seq results from differential expression analysis between H3aclow/H3achigh glioblastomas. Table S2: Complete RNA-seq results from differential expression analysis between TSA/untreated cultures.

## References

[1] Louis D.N., Perry A., Wesseling P., Brat D.J., Cree I.A., Figarella-Branger D., Hawkins C., Ng H.K., Pfister S.M., Reifenberger G. (2021). The 2021 WHO Classification of Tumors of the Central Nervous System: A summary. Neuro-Oncology.

[2] Stupp R., Mason W.P., van den Bent M.J., Weller M., Fisher B., Taphoorn M.J., Belanger K., Brandes A.A., Marosi C., Bogdahn U. (2005). Radiotherapy plus concomitant and adjuvant temozolomide for glioblastoma. N. Engl. J. Med..

[3] Valor L.M., Hervas-Corpion I. (2020). The Epigenetics of Glioma Stem Cells: A Brief Overview. Front. Oncol..

[4] Weller M., Stupp R., Reifenberger G., Brandes A.A., van den Bent M.J., Wick W., Hegi M.E. (2010). MGMT promoter methylation in malignant gliomas: Ready for personalized medicine?. Nat. Rev. Neurol..

[5] Turcan S., Rohle D., Goenka A., Walsh L.A., Fang F., Yilmaz E., Campos C., Fabius A.W., Lu C., Ward P.S. (2012). IDH1 mutation is sufficient to establish the glioma hypermethylator phenotype. Nature.

[6] Chen R., Zhang M., Zhou Y., Guo W., Yi M., Zhang Z., Ding Y., Wang Y. (2020). The application of histone deacetylases inhibitors in glioblastoma. J. Exp. Clin. Cancer Res..

[7] Kim M.S., Blake M., Baek J.H., Kohlhagen G., Pommier Y., Carrier F. (2003). Inhibition of histone deacetylase increases cytotoxicity to anticancer drugs targeting DNA. Cancer Res..

[8] Chinnaiyan P., Cerna D., Burgan W.E., Beam K., Williams E.S., Camphausen K., Tofilon P.J. (2008). Postradiation sensitization of the histone deacetylase inhibitor valproic acid. Clin. Cancer Res..

[9] Krauze A.V., Megan M., Theresa C.Z., Peter M., Shih J.H., Tofilon P.J., Rowe L., Gilbert M., Camphausen K. (2020). The addition of Valproic acid to concurrent radiation therapy and temozolomide improves patient outcome: A Correlative analysis of RTOG 0525, SEER and a Phase II NCI trial. Cancer Stud. Ther..

[10] Kamitani H., Taniura S., Watanabe K., Sakamoto M., Watanabe T., Eling T. (2002). Histone acetylation may suppress human glioma cell proliferation when p21 WAF/Cip1 and gelsolin are induced. Neuro-Oncology.

[11] Wang Z.M., Hu J., Zhou D., Xu Z.Y., Panasci L.C., Chen Z.P. (2002). Trichostatin A inhibits proliferation and induces expression of p21WAF and p27 in human brain tumor cell lines. Ai Zheng.

[12] Yin D., Ong J.M., Hu J., Desmond J.C., Kawamata N., Konda B.M., Black K.L., Koeffler H.P. (2007). Suberoylanilide hydroxamic acid, a histone deacetylase inhibitor: Effects on gene expression and growth of glioma cells in vitro and in vivo. Clin. Cancer Res..

[13] Mottet D., Pirotte S., Lamour V., Hagedorn M., Javerzat S., Bikfalvi A., Bellahcene A., Verdin E., Castronovo V. (2009). HDAC4 represses p21(WAF1/Cip1) expression in human cancer cells through a Sp1-dependent, p53-independent mechanism. Oncogene.

[14] Wu Q., Berglund A.E., Etame A.B. (2021). The Impact of Epigenetic Modifications on Adaptive Resistance Evolution in Glioblastoma. Int. J. Mol. Sci..

[15] Hervouet E. (2018). The Promising Role of New Generation HDACis in Anti-Cancer Therapies. Ebiomedicine.

[16] Williams M.J., Singleton W.G., Lowis S.P., Malik K., Kurian K.M. (2017). Therapeutic Targeting of Histone Modifications in Adult and Pediatric High-Grade Glioma. Front. Oncol..

[17] Kitange G.J., Mladek A.C., Carlson B.L., Schroeder M.A., Pokorny J.L., Cen L., Decker P.A., Wu W., Lomberk G.A., Gupta S.K. (2012). Inhibition of histone deacetylation potentiates the evolution of acquired temozolomide resistance linked to MGMT upregulation in glioblastoma xenografts. Clin. Cancer Res..

[18] Yu Z.Q., Zhang B.L., Ni H.B., Liu Z.H., Wang J.C., Ren Q.X., Mo J.B., Xiong Y., Yao R.Q., Gao D.S. (2014). Hyperacetylation of histone H3K9 involved in the promotion of abnormally high transcription of the gdnf gene in glioma cells. Mol. Neurobiol..

[19] Ku M.C., Wolf S.A., Respondek D., Matyash V., Pohlmann A., Waiczies S., Waiczies H., Niendorf T., Synowitz M., Glass R. (2013). GDNF mediates glioblastoma-induced microglia attraction but not astrogliosis. Acta Neuropathol..

[20] Yang W., Xia Y., Hawke D., Li X., Liang J., Xing D., Aldape K., Hunter T., Alfred Yung W.K., Lu Z. (2012). PKM2 phosphorylates histone H3 and promotes gene transcription and tumorigenesis. Cell.

[21] Yang W., Xia Y., Ji H., Zheng Y., Liang J., Huang W., Gao X., Aldape K., Lu Z. (2011). Nuclear PKM2 regulates beta-catenin transactivation upon EGFR activation. Nature.

[22] Liu B.L., Cheng J.X., Zhang X., Wang R., Zhang W., Lin H., Xiao X., Cai S., Chen X.Y., Cheng H. (2010). Global histone modification patterns as prognostic markers to classify glioma patients. Cancer Epidemiol. Biomark. Prev..

[23] Hervas-Corpion I., Gallardo-Orihuela A., Catalina-Fernandez I., Iglesias-Lozano I., Soto-Torres O., Geribaldi-Doldan N., Dominguez-Garcia S., Luna-Garcia N., Romero-Garcia R., Mora-Lopez F. (2021). Potential Diagnostic Value of the Differential Expression of Histone H3 Variants between Low- and High-Grade Gliomas. Cancers.

[24] Aryee M.J., Jaffe A.E., Corrada-Bravo H., Ladd-Acosta C., Feinberg A.P., Hansen K.D., Irizarry R.A. (2014). Minfi: A flexible and comprehensive Bioconductor package for the analysis of Infinium DNA methylation microarrays. Bioinformatics.

[25] Assenov Y., Muller F., Lutsik P., Walter J., Lengauer T., Bock C. (2014). Comprehensive analysis of DNA methylation data with RnBeads. Nat. Methods.

[26] Ritchie M.E., Phipson B., Wu D., Hu Y., Law C.W., Shi W., Smyth G.K. (2015). limma powers differential expression analyses for RNA-sequencing and microarray studies. Nucleic Acids Res..

[27] Rabaneda L.G., Carrasco M., Lopez-Toledano M.A., Murillo-Carretero M., Ruiz F.A., Estrada C., Castro C. (2008). Homocysteine inhibits proliferation of neuronal precursors in the mouse adult brain by impairing the basic fibroblast growth factor signaling cascade and reducing extracellular regulated kinase 1/2-dependent cyclin E expression. FASEB J..

[28] Pollard S.M., Yoshikawa K., Clarke I.D., Danovi D., Stricker S., Russell R., Bayani J., Head R., Lee M., Bernstein M. (2009). Glioma stem cell lines expanded in adherent culture have tumor-specific phenotypes and are suitable for chemical and genetic screens. Cell Stem Cell.

[29] Alenda C., Rojas E., Valor L.M. (2021). FFPE samples from cavitational ultrasonic surgical aspirates are suitable for RNA profiling of gliomas. PLoS ONE.

[30] Medvedeva Y.A., Lennartsson A., Ehsani R., Kulakovskiy I.V., Vorontsov I.E., Panahandeh P., Khimulya G., Kasukawa T., Drablos F. (2015). EpiFactors: A comprehensive database of human epigenetic factors and complexes. Database.

[31] Grabowicz I.E., Wilczynski B., Kaminska B., Roura A.J., Wojtas B., Dabrowski M.J. (2021). The role of epigenetic modifications, long-range contacts, enhancers and topologically associating domains in the regulation of glioma grade-specific genes. Sci. Rep..

[32] Deaton A.M., Bird A. (2011). CpG islands and the regulation of transcription. Genes Dev..

[33] Stepniak K., Machnicka M.A., Mieczkowski J., Macioszek A., Wojtas B., Gielniewski B., Poleszak K., Perycz M., Krol S.K., Guzik R. (2021). Mapping chromatin accessibility and active regulatory elements reveals pathological mechanisms in human gliomas. Nat. Commun..

[34] Aras Y., Erguven M., Aktas E., Yazihan N., Bilir A. (2016). Antagonist activity of the antipsychotic drug lithium chloride and the antileukemic drug imatinib mesylate during glioblastoma treatment in vitro. Neurol. Res..

[35] Sabanciota P.A., Erguven M., Yaziotahan N., Aktas E., Aras Y., Civelek E., Aydoseli A., Imer M., Gurtekin M., Bilir A. (2014). Sorafenib and lithium chloride combination treatment shows promising synergistic effects in human glioblastoma multiforme cells in vitro but midkine is not implicated. Neurol. Res..

[36] Erguven M., Bilir A., Yazihan N., Ermis E., Sabanci A., Aktas E., Aras Y., Alpman V. (2011). Decreased therapeutic effects of noscapine combined with imatinib mesylate on human glioblastoma in vitro and the effect of midkine. Cancer Cell Int..

[37] He M., Chen X., Luo M., Ouyang L., Xie L., Huang Z., Liu A. (2020). Suppressor of cytokine signaling 1 inhibits the maturation of dendritic cells involving the nuclear factor kappa B signaling pathway in the glioma microenvironment. Clin. Exp. Immunol..

[38] Ogden A.T., Horgan D., Waziri A., Anderson D., Louca J., McKhann G.M., Sisti M.B., Parsa A.T., Bruce J.N. (2006). Defective receptor expression and dendritic cell differentiation of monocytes in glioblastomas. Neurosurgery.

[39] Idbaih A., Carvalho Silva R., Criniere E., Marie Y., Carpentier C., Boisselier B., Taillibert S., Rousseau A., Mokhtari K., Ducray F. (2008). Genomic changes in progression of low-grade gliomas. J. Neurooncol..

[40] Belotti Y., Tolomeo S., Yu R., Lim W.T., Lim C.T. (2022). Prognostic Neurotransmitter Receptors Genes Are Associated with Immune Response, Inflammation and Cancer Hallmarks in Brain Tumors. Cancers.

[41] Yang J., Yang Q. (2020). Identification of Core Genes and Screening of Potential Targets in Glioblastoma Multiforme by Integrated Bioinformatic Analysis. Front. Oncol..

[42] Yu Z., Du M., Lu L. (2022). A Novel 16-Genes Signature Scoring System as Prognostic Model to Evaluate Survival Risk in Patients with Glioblastoma. Biomedicines.

[43] Xue W., Chen J., Liu X., Gong W., Zheng J., Guo X., Liu Y., Liu L., Ma J., Wang P. (2018). PVT1 regulates the malignant behaviors of human glioma cells by targeting miR-190a-5p and miR-488-3p. Biochim. Biophys. Acta Mol. Basis Dis..

[44] Feyissa A.M., Carrano A., Wang X., Allen M., Ertekin-Taner N., Dickson D.W., Jentoft M.E., Rosenfeld S.S., Tatum W.O., Ritaccio A.L. (2021). Analysis of intraoperative human brain tissue transcriptome reveals putative risk genes and altered molecular pathways in glioma-related seizures. Epilepsy Res..

[45] Lin B., Lee H., Yoon J.G., Madan A., Wayner E., Tonning S., Hothi P., Schroeder B., Ulasov I., Foltz G. (2015). Global analysis of H3K4me3 and H3K27me3 profiles in glioblastoma stem cells and identification of SLC17A7 as a bivalent tumor suppressor gene. Oncotarget.

[46] Gladitz J., Klink B., Seifert M. (2018). Network-based analysis of oligodendrogliomas predicts novel cancer gene candidates within the region of the 1p/19q co-deletion. Acta Neuropathol. Commun..

[47] Aruga J., Yokota N., Mikoshiba K. (2003). Human SLITRK family genes: Genomic organization and expression profiling in normal brain and brain tumor tissue. Gene.

[48] Zhou Y., Yang L., Zhang X., Chen R., Chen X., Tang W., Zhang M. (2019). Identification of Potential Biomarkers in Glioblastoma through Bioinformatic Analysis and Evaluating Their Prognostic Value. Biomed. Res. Int..

[49] Murphy K.A., Lechner M.G., Popescu F.E., Bedi J., Decker S.A., Hu P., Erickson J.R., O’Sullivan M.G., Swier L., Salazar A.M. (2012). An in vivo immunotherapy screen of costimulatory molecules identifies Fc-OX40L as a potent reagent for the treatment of established murine gliomas. Clin. Cancer Res..

[50] Lucio-Eterovic A.K., Cortez M.A., Valera E.T., Motta F.J., Queiroz R.G., Machado H.R., Carlotti C.G., Neder L., Scrideli C.A., Tone L.G. (2008). Differential expression of 12 histone deacetylase (HDAC) genes in astrocytomas and normal brain tissue: Class II and IV are hypoexpressed in glioblastomas. BMC Cancer.

[51] Hanisch D., Krumm A., Diehl T., Stork C.M., Dejung M., Butter F., Kim E., Brenner W., Fritz G., Hofmann T.G. (2022). Class I HDAC overexpression promotes temozolomide resistance in glioma cells by regulating RAD18 expression. Cell Death Dis..

[52] Dali-Youcef N., Froelich S., Moussallieh F.M., Chibbaro S., Noel G., Namer I.J., Heikkinen S., Auwerx J. (2015). Gene expression mapping of histone deacetylases and co-factors, and correlation with survival time and 1H-HRMAS metabolomic profile in human gliomas. Sci. Rep..

[53] Li J., Yan X., Liang C., Chen H., Liu M., Wu Z., Zheng J., Dang J., La X., Liu Q. (2022). Comprehensive Analysis of the Differential Expression and Prognostic Value of Histone Deacetylases in Glioma. Front. Cell Dev. Biol..

[54] Cohen A.L., Piccolo S.R., Cheng L., Soldi R., Han B., Johnson W.E., Bild A.H. (2013). Genomic pathway analysis reveals that EZH2 and HDAC4 represent mutually exclusive epigenetic pathways across human cancers. BMC Med. Genom..

[55] Marampon F., Megiorni F., Camero S., Crescioli C., McDowell H.P., Sferra R., Vetuschi A., Pompili S., Ventura L., De Felice F. (2017). HDAC4 and HDAC6 sustain DNA double strand break repair and stem-like phenotype by promoting radioresistance in glioblastoma cells. Cancer Lett..

[56] Liu Y., Duan X., Zhang C., Yuan J., Wen J., Zheng C., Shi J., Yuan M. (2022). KAT6B May Be Applied as a Potential Therapeutic Target for Glioma. J. Oncol..

[57] Wiesel-Motiuk N., Assaraf Y.G. (2020). The key roles of the lysine acetyltransferases KAT6A and KAT6B in physiology and pathology. Drug Resist. Updat..

[58] Klein B.J., Jang S.M., Lachance C., Mi W., Lyu J., Sakuraba S., Krajewski K., Wang W.W., Sidoli S., Liu J. (2019). Histone H3K23-specific acetylation by MORF is coupled to H3K14 acylation. Nat. Commun..

[59] Lewis P.W., Muller M.M., Koletsky M.S., Cordero F., Lin S., Banaszynski L.A., Garcia B.A., Muir T.W., Becher O.J., Allis C.D. (2013). Inhibition of PRC2 activity by a gain-of-function H3 mutation found in pediatric glioblastoma. Science.

[60] An S., Camarillo J.M., Huang T.Y., Li D., Morris J.A., Zoltek M.A., Qi J., Behbahani M., Kambhampati M., Kelleher N.L. (2020). Histone tail analysis reveals H3K36me2 and H4K16ac as epigenetic signatures of diffuse intrinsic pontine glioma. J. Exp. Clin. Cancer Res..

[61] Gallo M., Coutinho F.J., Vanner R.J., Gayden T., Mack S.C., Murison A., Remke M., Li R., Takayama N., Desai K. (2015). MLL5 Orchestrates a Cancer Self-Renewal State by Repressing the Histone Variant H3.3 and Globally Reorganizing Chromatin. Cancer Cell.

[62] Riva G., Butta V., Cilibrasi C., Baronchelli S., Redaelli S., Dalpra L., Lavitrano M., Bentivegna A. (2016). Epigenetic targeting of glioma stem cells: Short-term and long-term treatments with valproic acid modulate DNA methylation and differentiation behavior, but not temozolomide sensitivity. Oncol. Rep..

