# Supplementary material for: Defining a Correlative Transcriptional Signature Associated with Bulk Histone H3 Acetylation Levels in Adult Glioblastomas
Source: Cells. 2023 Jan 19;12(3):374. doi: 10.3390/cells12030374 (PMC9913072; doi:10.3390/cells12030374)
Supplement: Supplementary file 1 [file cells-12-00374-s001.zip › cells-2153571 - supplementary.pdf]

Supplementary material: Defining a correlative transcriptional signature associated with bulk histone H3 acetylation levels in adult glioblastomas

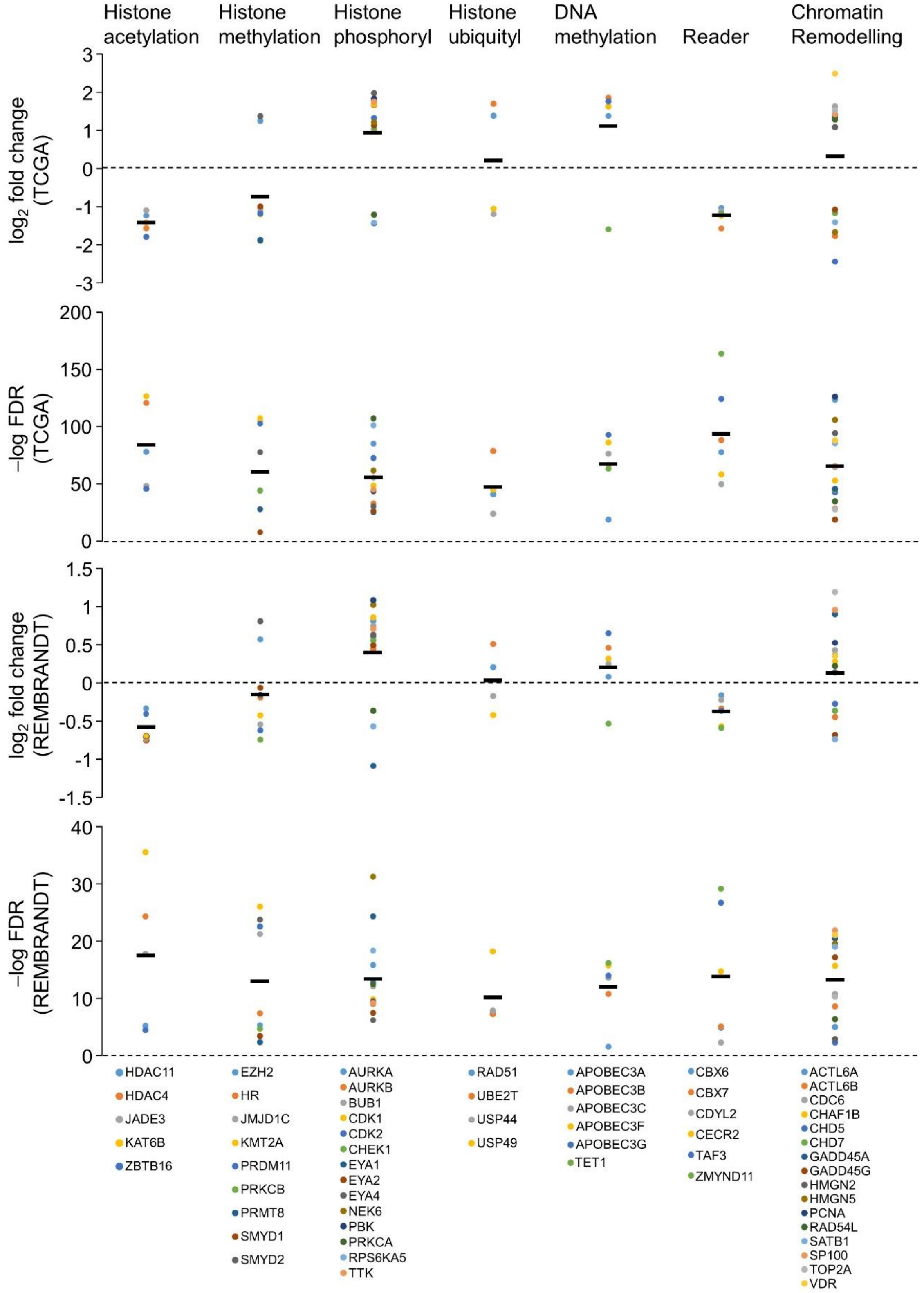

Figure S1. Differential expression changes of epigenetic-related genes in TCGA and REMBRANDT gliomas: Genes which differential expression between the datasets of "GBM" and "LGG" projects of TCGA that were also confirmed in the REMBRANDT cohort. FDR, false discovery rate.

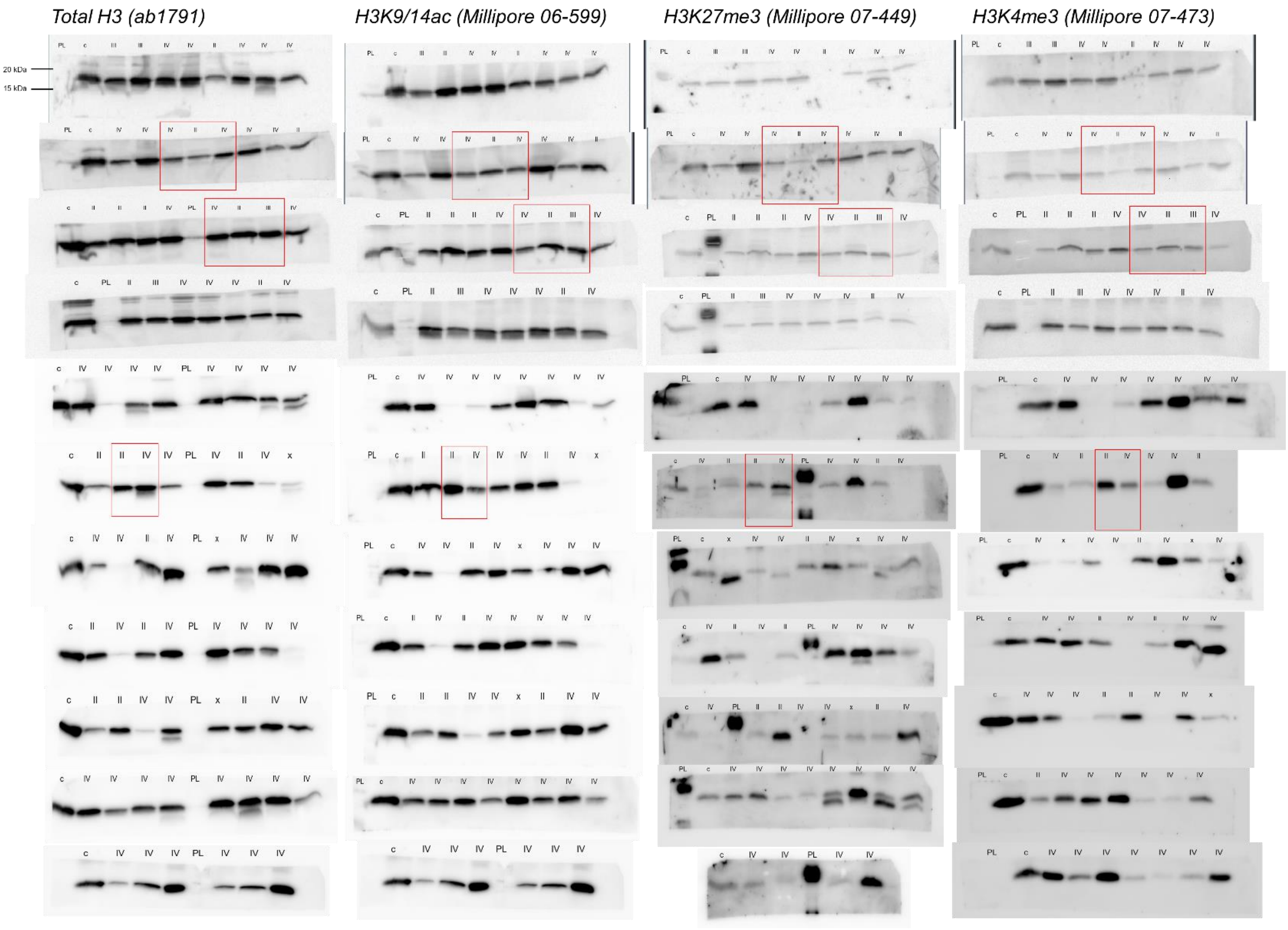

Figure S2. Raw western blots of histone H3 modifications in glioma resections: PL, protein ladder; x, discarded sample (doubtful diagnosis). c, the same non-related sample (mouse cerebellum) used as a reference for interblots normalization (ref. 23 of main text). Red squares indicate the samples that were used for the image crops shown in Figure 1C.

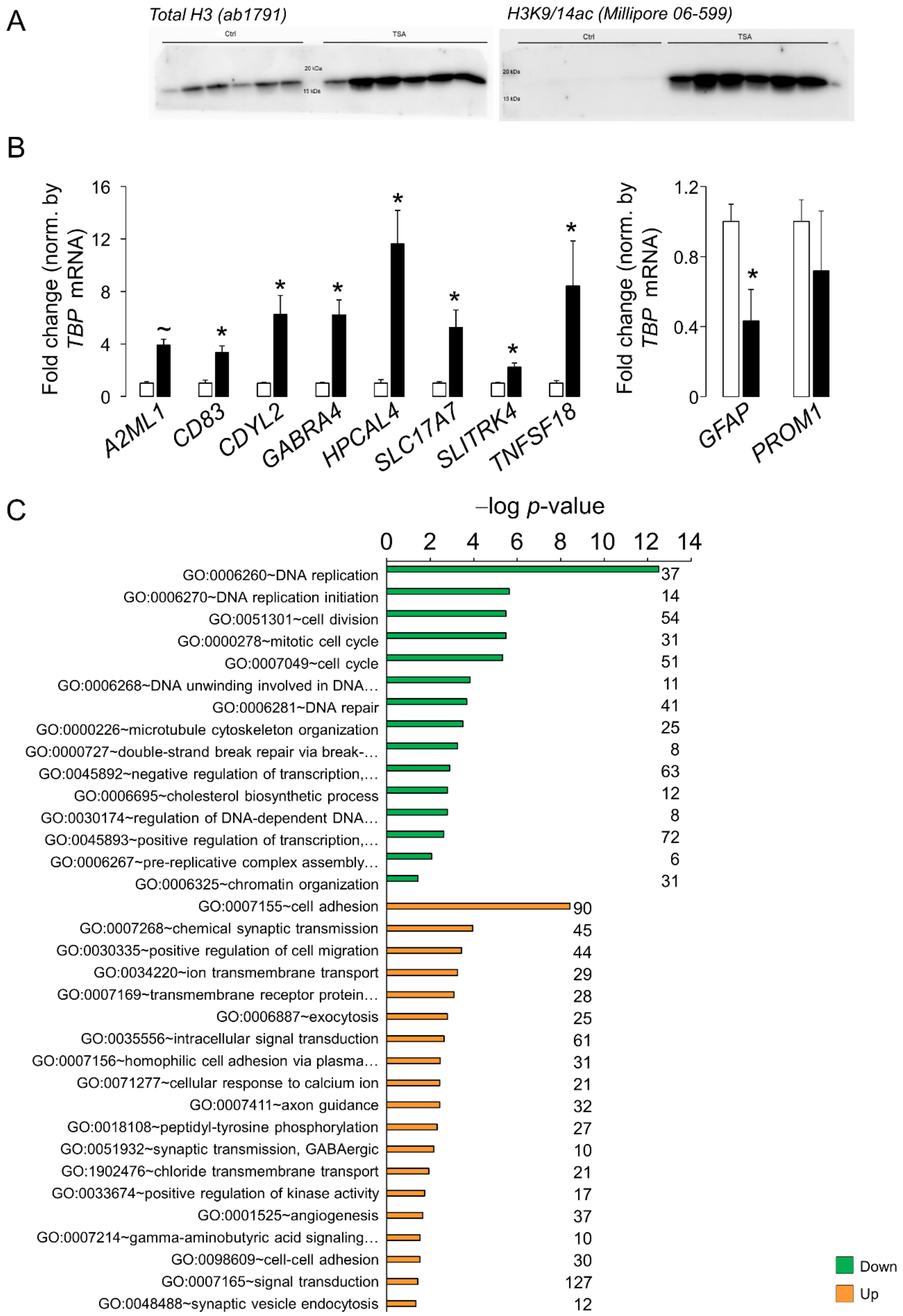

Figure S3. Histone H3 acetylation in TSA-treated glioma culture: (**a**) Raw western blots of histone H3 acetylation in glioma cultures treated with trichostatin A (TSA). (**b**) Validation of the RNA-seq by RT-qPCR assays in independent cultures. Data are expressed as the mean ±SEM. n = 4 for each condition. Left panel, upregulated genes in the RNA-seq analysis; right panel, downregulated (GFAP)

and nonchanging genes (PROM1/CD133). ~, p-value < 0.1; *, p-value < 0.05, Mann-Whiteny U-test between both groups. (**c**) Significantly enriched Gene Ontology (GO) terms (FDR or false Discovery rate < 0.05, DAVID) in the differentially expressed genes between TSA and control cultures, represented as -log adjusted p-value (bars) and number of genes (besides the bars).
